# Comparison of soft tissue simulations between two planning software programs for orthognathic surgery

**DOI:** 10.1038/s41598-022-08991-7

**Published:** 2022-03-23

**Authors:** Ali Modabber, Tanja Baron, Florian Peters, Kristian Kniha, Golamreza Danesh, Frank Hölzle, Nassim Ayoub, Stephan Christian Möhlhenrich

**Affiliations:** 1 Department of Oral and Maxillofacial Surgery, University Hospital of Aachen, Pauwelsstraße 30, 52074 Aachen, Germany; 2 Department of Orthodontics, University of Witten/Herdecke, Alfred-Herrhausen Str. 45, 58455 Witten, Germany

**Keywords:** Software, Outcomes research, Musculoskeletal system

## Abstract

The aim of this study was to compare the soft tissue predicative abilities of two established programs depending on the surgical technique and amount of displacement. On the basis of 50 computed tomography images, 11 orthognathic operations with differences in displacement distances and technique (maxillary advancement, MxA; maxillary impaction, MxI; mandibular setback, MnS; mandibular advancement, MnA bimaxillary displacement, MxA/MnS) as well as corresponding soft tissue predictions were simulated using the programs Dolphin (D) and ProPlan (PP). For all the soft tissue predictions by the two programs, eight linear and two angular measurements were performed and compared. The simulation of maxillary impaction showed a similar soft tissue behaviour between the two programs. However, differences or divergent behaviours were observed for other procedures. In the middle third of the face these significant differences concerned in particular the nasolabial angle (Ns-Sn-Ls)(5 mm-MA, D: 119.9 ± 8.6° vs. PP: 129.5 ± 8.4°; 7 mm-MnS: D: 128.5 ± 8.2° vs. PP: 129.6 ± 8.1°; 10 mm-MnA D: 126.0 ± 8.0° vs. PP: 124.9 ± 8.4°; 5 mm-MxA/4 mm-MnS, D: 120.2 ± 8.7° vs. PP: 129.9 ± 8.3°; all p < 0.001) and in the lower third the mentolabial angle (Pog´-B´-Li) (5 mm-MA, D: 133.2 ± 11.4° vs. PP: 126.8 ± 11.6°; 7 mm-MnS: D: 133.1 ± 11.3° vs. PP: 124.6 ± 11.9°; 10 mm-MnA D: 133.3 ± 11.5° vs. PP: 146.3 ± 11.1°; bignathic 5 mm-MxA/4 mm-MnS, D: 133.1 ± 11.4° vs. PP: 122.7 ± 11.9°; all p < 0.001) and the distance of the inferior lip to the aesthetic Line (E-Line-Li) (5 mm-MA, D: 3.7 ± 2.3 mm vs. PP: 2.8 ± 2.5 mm; 7 mm-MnS: D: 5.1 ± 3.0 mm vs. PP: 3.3 ± 2.3 mm; 10 mm-MnA D: 2.5 ± 1.6 mm vs. PP: 3.9 ± 2.8 mm; bignathic 5 mm-MxA/4 mm-MnS, D: 4.8 ± 3.0 mm vs. PP: 2.9 ± 2.0 mm; all p < 0.001). The soft tissue predictions by the tested programs differed in simulation outcome, which led to the different, even divergent, results. However, the significant differences are often below a clinically relevant level. Consequently, soft tissue prediction must be viewed critically, and its actual benefit must be clarified.

## Introduction

Three-dimensional computer planning has been established in orthognathic surgery owing to its valuable roles in surgical planning, operative outcome assessment and patient communication^1–3^. The modern surgical planning software allows pre-operative simulation of the soft tissue behaviour in the context of orthognathic surgery. Therefore, radiological imaging techniques are used to generate virtual models of patients' facial skulls and associated soft tissues. Afterwards, the operation can be virtually performed. Then, the post-operative outcome is simulated, and the simulation should illustrate the predicted aesthetic appearance to the surgeon and the patient^4^. Among the benefits of the simulation are that it allows the surgeon to experience different surgical scenarios and assess the corresponding surgery, including the anatomy, from all three spatial planes. Finally, digital surgical planning should provide significant benefits for the surgeon in terms of treatment time, precision and minimisation of errors and complications^5^.

Different surgical programs with soft tissue simulation tools are available for three-dimensional planning of orthognathic surgery. The software differs with regard to its soft tissue prediction according to the underlying physical model. Sparse models require landmarking and rely on interpolation between points, whereas others programs used dense volumetric models such as finite element, mass spring or tensor models, which need a volumetric tetrahedral mesh containing all the facial tissues^6^. However, these programs are known to have a soft tissue prediction error of < 2 mm. Whether this deviation is clinically relevant is controversial^7–12^. These discrepancies can be due to the underlying algorithm or pre-operative planning and the different surgical outcome achieved in real life. In this context, outcome inaccuracies of 0.99 or 1.17 mm between planned and performed mono- and bi-maxillary orthognathic surgeries have been reported^13,14^.

Recently, Knoops et al. evaluated three different programs with regard three-dimensional soft tissue prediction in seven patients who received Le Fort I maxillary advancement^15^. They investigated the features and limitations of the three different soft tissue prediction programs, namely Dolphin Imaging (Dolphin & Management Solution, Chatsworth, CA, USA), ProPlan CMF (Materialise NV, Leuven, Belgium) and a probabilistic finite element method (PFEM) to determine how their limitations may affect their clinical usefulness for Le Fort I osteotomies. The focal points of interest were set on the upper lip and paranasal regions.

They reported that Dolphin, which uses a landmark-based algorithm for patient-specific bone-to-soft tissue ratios, works well for cephalometric radiography but was limited with regard to its three-dimensional accuracy^15^. By contrast, ProPlan and PFEM provide better three-dimensional predictions with greater displacements of the underlying bony structures. Furthermore, PFEM allows for defining patient- or population-specific material properties, whereas ProPlan allows for no adjustments of soft tissue parameters. Knoops et al. concluded that the topological discrepancies in predictions were due to the differences between the three algorithms, the non-negligible influence of the mismatch between the planned and post-operative maxillary positions and the learning curve associated with sophisticated programs such as PFEM^15^.

This shows that currently, no uniform principle for soft tissue simulation exists; thus, no generally valid result can be generated. This is unfavourable with regard to the high expectations of patients from the pre-operative simulation of possible facial changes. Therefore, the aim of the present investigation was to compare the possible differences in soft tissue simulation between the two established programs, Dolphin and ProPlan, depending on different displacement directions and distances.

The null hypothesis of the present study was that there would be no differences between the two soft tissue simulations when the same surgical procedure was present and the same displacement distance.

## Materials and methods

This investigation was approved the Ethics Committee of the Medical Faculty of the RWTH Aachen, Germany (EK 231/17) and performed in accordance with the Declaration of Helsinki. A general consent for data processing was given from all participants for scientific research.

In this study, virtual mono- and bi-maxillary orthognathic surgical procedures were simulated on the basis of 50 computed tomography (CT) images obtained using a 128-row multi-slice CT scanner Somatom Definition Flash (Siemens, Munich, Germany) from the clinic's internal radiological database. The surgery was planned using the software programs ProPlan CMF v3.0.1.5 (Materialise NV, Leuven, Belgium) and Dolphin Imaging v11.09.07.24 Premium (Dolphin & Management Solution, Chatsworth, CA).

Inclusion in the study was based on the following criteria: patients without fractures of the viscerocranium, deformities in facial soft tissues or insufficient dentition or metal restorations that led to associated artifacts. Furthermore, all the patients were verified to have full occlusion with habitual lip closure.

The experimental group consisted of 29 male and 21 female patients ranging in age from 16 to 56 years. The mean (± SD) age at the time of CT scan was 32.9 ± 9.5 years. The data sets were generated from 2012 to 2017. In terms of phenotype, an attempt was made to represent the average European and Anglo ethnicities. African and Asian characteristics were excluded owing to peculiarities of the ethnic groups.

### Segmentation process

**Figure 1:**
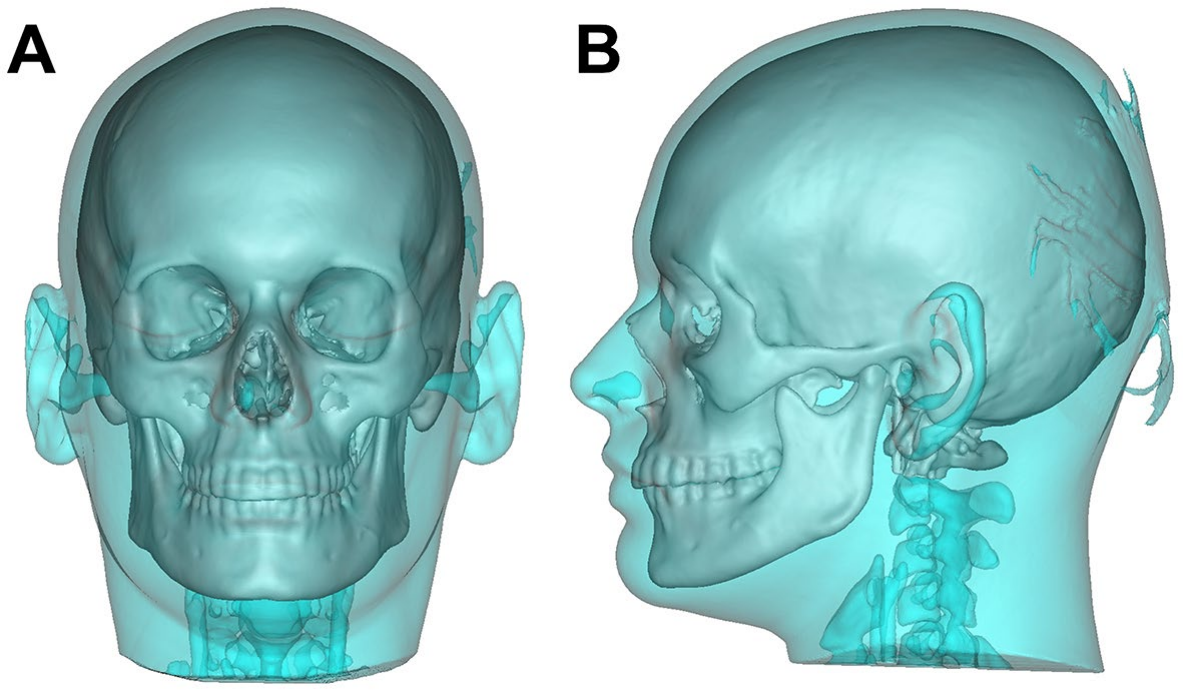
Segmented models of the facial soft tissue and the cranial and facial skeleton including the maxilla and the mandible.

Segmentation was performed using the surgical planning software ProPlan with a defined Hounsfield scale for hard and soft tissues. First, a coarsely segmented model was created fully automatically, which was next cleaned of any artifacts by manual fine segmentation. The result of the segmentation was a three-part model consisting of a mask of the facial soft tissue (1), the cranial and facial skeleton including the maxilla (2) and the mandible (3) (Fig. 1). Afterwards, the data set was then imported as an STL file into the second planning software, Dolphin. This ensured identical soft and hard tissues in both planning programs.

### Virtual orthognathic surgery and soft tissue simulation

For each data set, 11 different displacement scenarios with their corresponding osteotomies were performed in the ProPlan and Dolphin software (Table 1). The osteotomy tools in both programs were used for this purpose. The thickness of the bone cut was set to 0.5 mm.

**Table 1:** Overview of the 11 different orthognathic surgery simulations per data set.

Orthognathic surgery	Procedure	Extent of displacement (mm)	Number
Monomaxillary	Mandibular setback	7	1
		4	2
	Mandibular advancement	4	3
		7	4
		10	5
	Maxillary advancement	3	6
		5	7
	Maxillary impaction	2	8
		5	9
Bimaxillary	Maxilla advancement	3	10
	Mandible setback	4	
	Maxilla advancement	2	11
	Maxilla impaction	5	
	Mandible setback	4	

**Figure 2:**
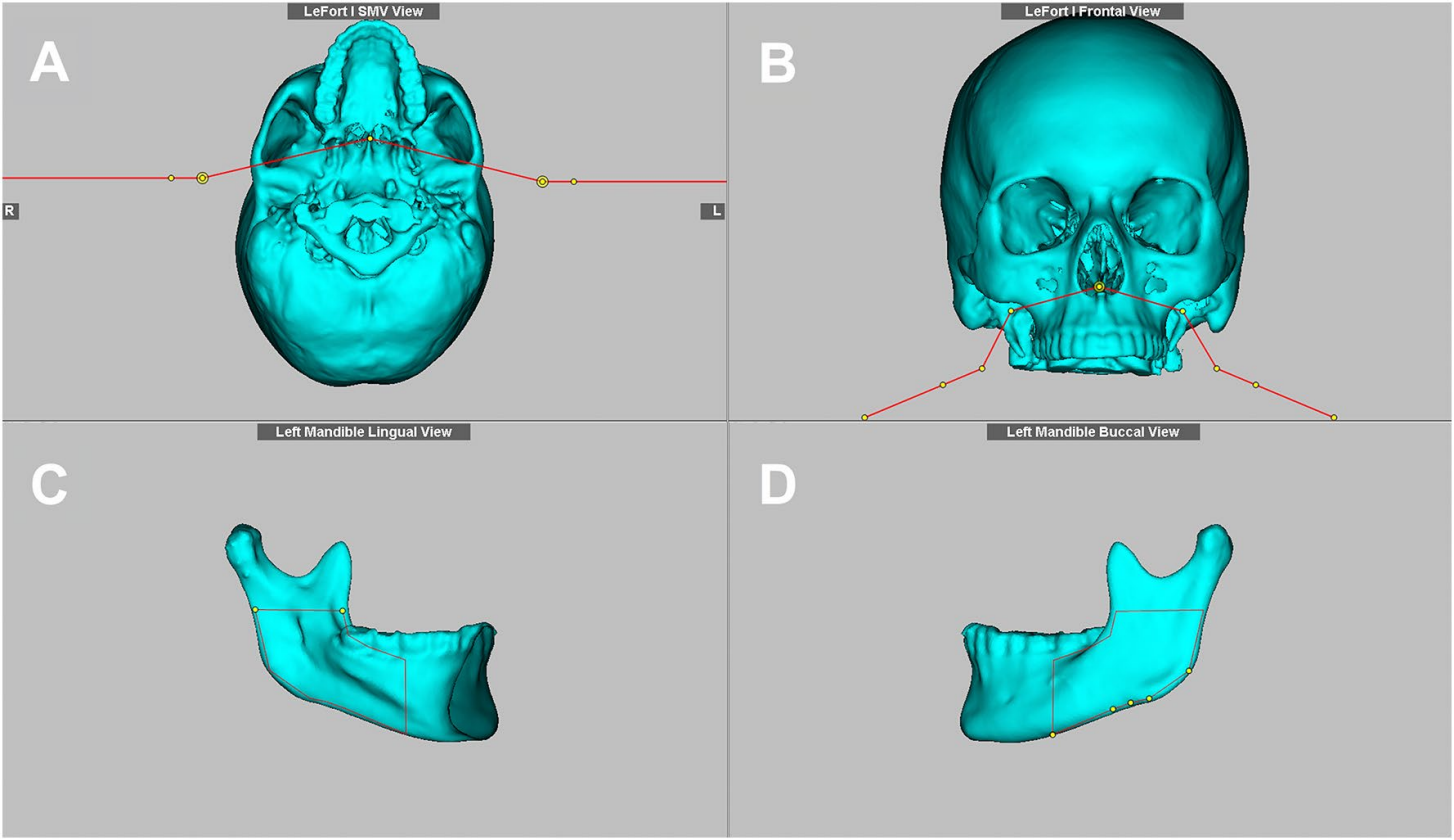
Osteotomy lines (red) for LeFort I from a submentovertex (**A**) and frontal view (**B**), and for mandibular sagittal split from a lingual (**C**) and buccal view (**D**) using Dolphin imaging.

The maxillary displacement simulation was based on a virtual Le Fort I osteotomy. This was performed using an osteotomy of the nasal septum from the anterior to the posterior nasal spine, horizontal osteotomies of the median and lateral maxillary sinus walls from the piriform aperture through the zygomatic buttress to the pterygo-maxillary and a posterior osteotomy through the pterygomaxillary connection (Fig. 2A,B). These osteotomies were planned above the level of the maxillary sinus floor at a distance approximately 5 mm to the apices of the teeth.

**Figure 3:**
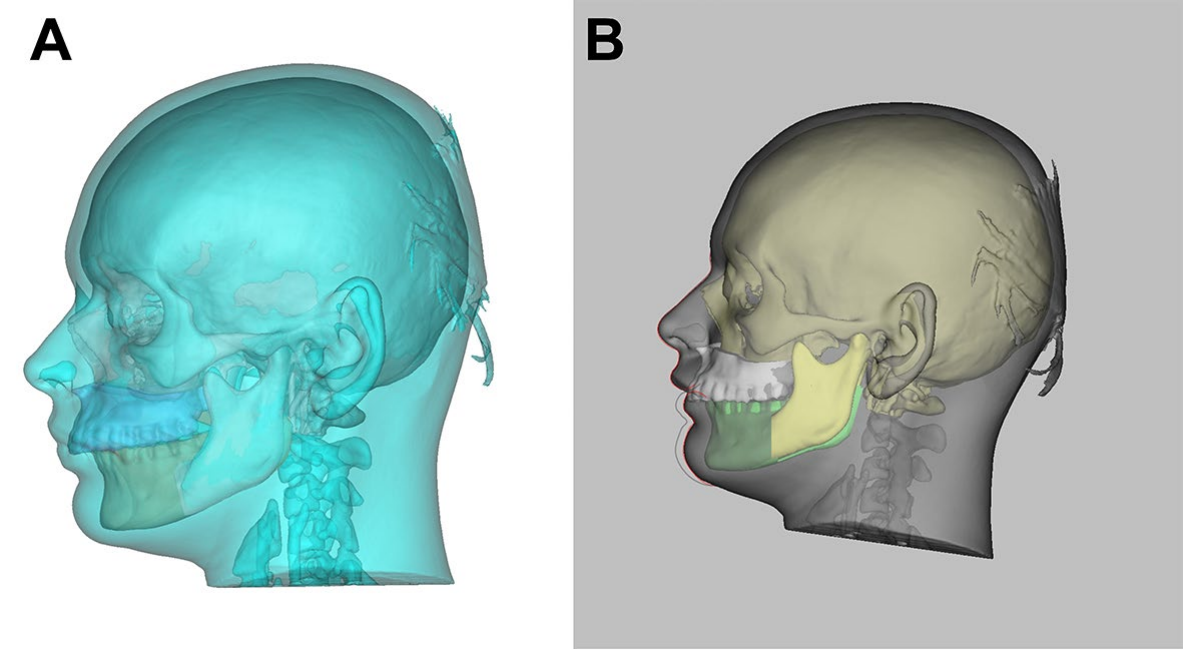
Profile view of the soft tissue simulation for the 7 mm mandibular setback using ProPlan CMF (**A**) and Dolphin imaging (**B**).

The displacement of the mandible was performed according to the Obwegeser/Dal Pont osteotomy technique. Therefore, a lingual osteotomy, which runs posterior to the mandibular foramen; a buccal bone cut that vertically passes the bone in the region of the first and second molars; and a third connecting osteotomy along the oblique line between the two osteotomies were simulated (Fig. 2C,D). Subsequently, the soft tissue simulation was performed fully automatically in both surgical planning software programs (Fig. 3).

### Soft tissue analysis

**Figure 4:**
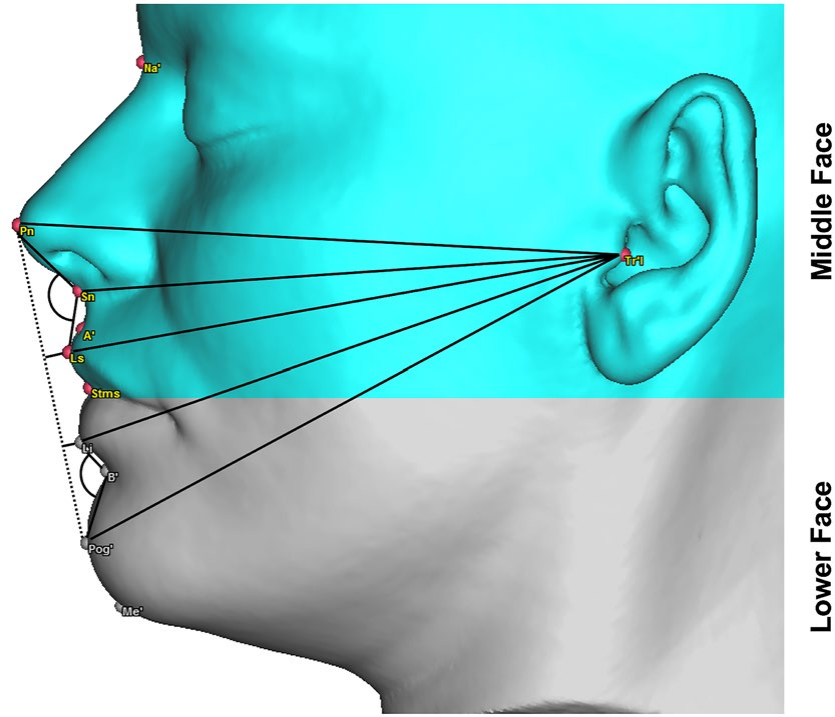
Angular and linear measurements of the middle (green) and lower (grey) third of the face in the profile view.

To ensure uniform measurements and prevent measurement errors, the soft tissue simulations from the two programs were analysed only in the ProPlan software. For this purpose, the simulated soft tissue from the Dolphin software was exported as an STL file to ProPlan. Afterwards, the defined landmarks were set on all 1,100 soft tissue data sets for soft tissue evaluation (Table 2). On the basis of these landmarks, eight linear and two angular representative measurements were performed (Fig. 4, Table 3). Thus, the Tragion (Tr) was used as one of the main landmarks, due this anatomical structure has already been shown in previous investigations to be stable and well recognisable^16–18^.

**Table 2:** Landmarks for the facial analysis.

Landmark	Abbreviation	Description
Alar curvature sulcus (right/left)	Scal (r/l)	The point located at the facial insertion of each alar base
Pronasale	Pn	The most anterior midpoint of the nasal tip
Subnasale	Sn	The midpoint on the nasolabial soft tissue contour between the columella crest and the upper lip
Labiale superius	Ls	The midpoint of the vermilion line of the upper lip
Labiale inferius	Li	The midpoint of the vermilion line of the lower lip
Soft tissue nasion	Ns	The midpoint on the soft tissue contour of the base of the nasal root, at the level of the frontonasal suture
Soft tissue pogonion	Pog ´	The most anterior midpoint of the chin
Soft tissue B point	B ´	The most posterior midpoint on the labiomental soft tissue contour that defines the border between the lower lip and the chin
Tragion	Tr	The point located at the upper margin of each tragus

**Table 3:** Angular and linear measurements of the middle and lower third of the face.

Abbreviation	Description
**Face middle third**	
Ns–Sn–Ls (°)	Nasolabial sulcus: angle from soft tissue nasion via subnasale to labiale superius
E-Line–Ls (mm)	Distance between Esthetic–Line and labiale superius
Tr–Ls (mm)	Distance between left tragion and labiale superius point
Tr–Pnʹ (mm)	Distance between left tragion and pronasale point
Tr–Sn (mm)	Distance between left tragion and subnasale point
Scal'r–Scal'l (mm)	Width of the base of the nose: distance between the right and left sulcus of the alar curvature
**Face lower third**	
Pog´–B´–Li (°)	Mentolabial sulcus: angle from point soft tissue pogonion via B point to labiale inferius
E-Line–Li (mm)	Distance between Esthetic–Line and labiale inferius
Tr–Li (mm)	Distance between left tragion and labiale inferius
Tr–Pog' (mm)	Distance between tragion and soft tissue pogonion

### Statistical analysis

The same investigator repeated the measurements after four weeks and the intraclass correlation coefficient (ICC) were assessed for calibration. Sufficient calibration was assumed ICC > 0.85 for all measurements and ranged overall between 0.88 and 0.92. The statistical analysis between the two groups was performed with GraphPad Prism V7.04 (GraphPad Software Inc., San Diego, CA, USA). Shapiro–Wilk test was applied to the data to confirm the presence of normal distribution. A two-way analysis of variance and post hoc Tukey test for multiple comparison were used to analyse the groups. In each analysis, Bonferroni adjustment was applied. The level of significance was set at *P* ≤ 0.05. All the results are expressed as mean ( ±) and standard deviation (SD).

## Results

**Figure 5:**
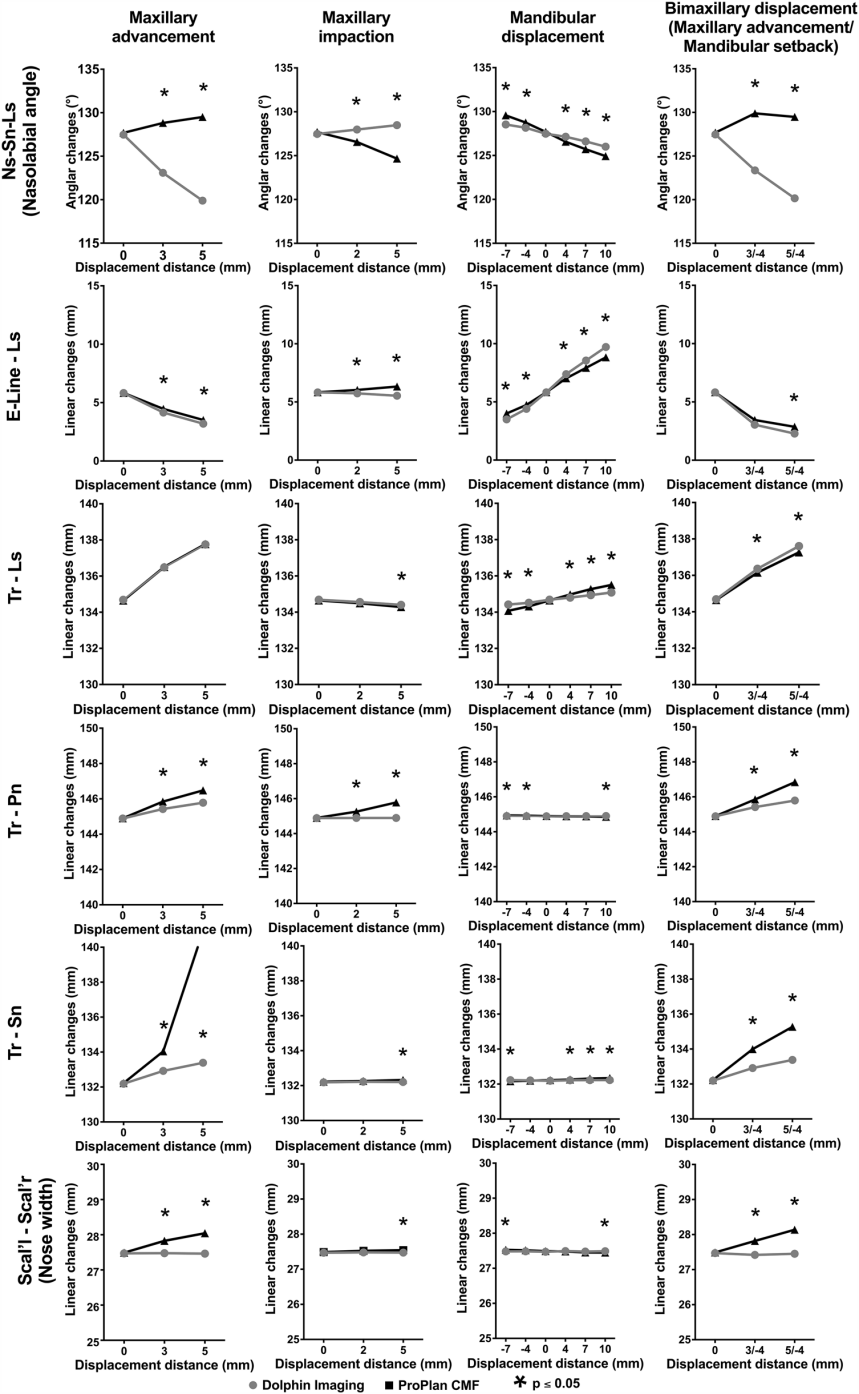
Line charts for the changes in the respective programs (ProPlan CMF vs. Dolphin imaging) in middle third of the face depending on surgical technique and displacement distance.

**Figure 6:**
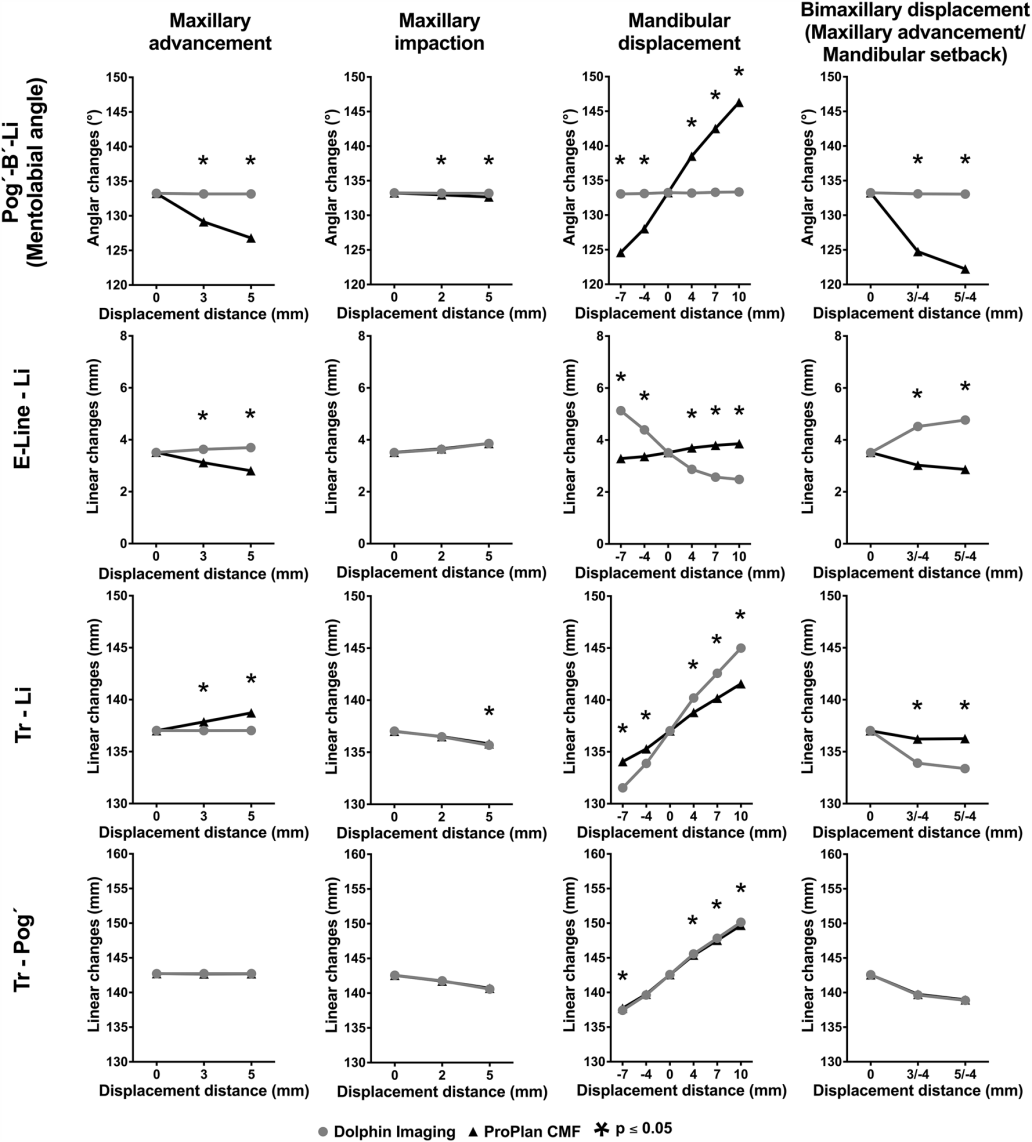
Line charts for the changes in the respective programs (ProPlan CMF vs. Dolphin imaging) in lower third of the face depending on surgical technique and displacement distance.

The mean values and SDs of the angular and linear changes in the middle and lower third of the face after all the orthognathic surgery simulations are shown in Figs. 5 and 6 and in the corresponding Tables 4, 5, 6, 7, 8. Furthermore, the corresponding p values of the comparison between the outcome of the planning programs Dolphin and ProPlan depending on the amount different displacement distances are presented. No significant differences were found between the soft tissue measurements in front of the surgical simulation using Dolphin and ProPlan (0 mm displacement distance: Dolphin vs. Problan). Thus, equal starting conditions in both groups can be assumed.

**Table 4:** Mean values, SDs and 95% confidence intervals of the angular and linear changes in the middle and lower third of the face after maxillary surgery simulation with the corresponding p values of the comparison between the outcome of the surgical planning programs Dolphin and ProPlan depending on the different maxillary advancement displacement distances.

	Displacement distance																	
	0 mm						3 mm						5 mm					
	Dolphin imaging		ProPlan CMF		MD	p-value	Dolphin imaging		ProPlan CMF		MD	p-value	Dolphin imaging		ProPlan CMF		MD	p-value
	Mean (SD)	95% CI	Mean (SD)	95% CI			Mean (SD)	95% CI	Mean (SD)	95% CI			Mean (SD)	95% CI	Mean (SD)	95% CI		
**Face middle third**																		
Ns–Sn–Ls	127.5 ± 8.2	125.2–129.8	127.7 ± 8.2	125.4–130.0	− 0.2	> 0.999	123.1 ± 8.4	120.7–125.5	128.8 ± 8.2	126.5–131.2	− 5.7	< 0.001	119.9 ± 8.6	117.4–122.3	129.5 ± 8.4	127.2–131.9	− 9.6	< 0.001
E-Line–Ls	5.8 ± 2.9	5.0–6.7	5.8 ± 2.9°	5.0–6.7	0.0	> 0.999	4.2 ± 2.6	3.4–4.9	4.5 ± 2.7	3.7–5.2	− 0.3	0.032	3.2 ± 2.3	2.5–3.9	3.5 ± 2.5	2.8–4.2	− 0.3	0.025
Tr–Ls	134.7 ± 6.7	132.8–136.6	134.6 ± 6.7	132.8–136.5	0.1	0.869	136.5 ± 6.7	134.6–138.4	136.5 ± 6.7	134.6–138.4	0.0	> 0.999	137.7 ± 6.7	135.8–139.7	137.8 ± 6.7	135.9–139.7	− 0.1	> 0.999
Tr–Pn´	144.9 ± 7.0	142.9–146.9	144.9 ± 7.0	142.9–146.9	0.0	> 0.999	145.4 ± 7.0	143.4–147.4	145.8 ± 7.1	143.8–147.9	− 0.4	< 0.001	145.8 ± 7.0	143.8–147.8	146.5 ± 7.1	144.5–148.5	1.3	< 0.001
Tr–Sn	132.2 ± 6.4	130.4–134.0	132.2 ± 6.5	130.4–134.1	0.0	> 0.999	132.9 ± 6.4	131.1–134.7	134.0 ± 6.5	132.2–135.9	− 1.1	< 0.001	133.4 ± 6.4	131.6–135.2	140.9 ± 6.8	139.0–142.8	− 7.5	< 0.001
Scal'r–Scal'l	27.5 ± 3.6	26.5–28.5	27.5 ± 3.6	26.5–28.5	0.0	> 0.999	27.5 ± 3.5	26.5–28.5	27.8 ± 3.6	26.8–28.9	− 0.3	< 0.001	27.5 ± 3.6	26.5–28.5	28.0 ± 3.6	27.0–29.1	− 0.5	< 0.001
**Face lower third**																		
Pog´–B´–Li	133.2 ± 11.4	130.0–136.5	133.2 ± 11.5	130.0–136.5	0.0	> 0.999	133.2 ± 11.4	129.9–136.4	129.1 ± 11.4	125.9 – 132.4	4.1	< 0.001	133.2 ± 11.4	129.9–136.4	126.8 ± 11.6	123.5–130.1	6.4	< 0.001
E-Line–Li	3.5 ± 2.6	2.7–4.2	3.5 ± 2.6	2.8–4.3	0.0	> 0.999	3.6 ± 2.6	2.9–4.4	3.1 ± 2.7	2.5–3.8	0.5	< 0.001	3.7 ± 2.3	3.0–4.5	2.8 ± 2.5	2.3–3.4	0.9	< 0.001
Tr – Li	137.0 ± 6.8	135.1–138.9	137.0 ± 6.8	135.1–138.9	0.0	> 0.999	137.0 ± 6.8	135.1–138.9	137.9 ± 6.8	135.9–139.8	− 0.9	< 0.001	137.0 ± 6.8	135.1–138.9	138.7 ± 6.8	136.8–140.7	− 1.7	< 0.001
Tr–Pog'	142.7 ± 7.8	140.4–145.0	142.6 ± 7.6	140.4–144.7	0.1	> 0.999	142.7 ± 7.8	140.4–145.0	141.7 ± 7.7	139.6–143.9	1.0	0.157	142.7 ± 7.8	140.4–145.0	140.7 ± 7.5	138.6–142.8	2.0	0.768

*SD* standard deviation, *CI* confidence interval, *MD* mean difference.

**Table 5:** Mean values, SDs and 95% confidence intervals of the angular and linear changes in the middle and lower third of the face after maxillary impaction simulation with the corresponding p values of the comparison between the outcome of the surgical planning programs Dolphin and ProPlan depending on different displacement distances.

	Displacement distance																	
	0 mm						2 mm						5 mm					
	Dolphin Imaging		ProPlan CMF		MD	p-value	Dolphin Imaging		ProPlan CMF		MD	P-value	Dolphin Imaging		ProPlan CMF		MD	p-value
	Mean (SD)	95% CI	Mean (SD)	95% CI			Mean (SD)	95% CI	Mean (SD)	95% CI			Mean (SD)	95% CI	Mean (SD)	95% CI		
**Face middle third**																		
Ns–Sn–Ls	127.5 ± 8.2	125.2–129.8	127.7 ± 8.2	125.4–130.0	− 0.2	> 0.999	128.0 ± 8.3	125.6–130.3	126.6 ± 8.3	124.2–128.9	1.4	< 0.001	128.5 ± 8.4	126.1–130.9	124.7 ± 8.5	122.2–127.1	3.8	< 0.001
E-Line–Ls	5.8 ± 2.9	5.0–6.7	5.8 ± 2.9	5.0–6.7	0.0	> 0.999	5.7 ± 2.9	4.9–6.6	6.0 ± 2.9	5.2–6.9	− 0.3	< 0.001	5.5 ± 2.9	4.7–6.4	6.3 ± 2.9	5.5–7.2	− 0.8	< 0.001
Tr–Ls	134.7 ± 6.7	132.8–136.6	134.6 ± 6.7	132.8–136.5	0.1	> 0.999	134.6 ± 6.7	132.7–136.5	134.5 ± 6.6	132.6–136.4	0.1	0.082	134.4 ± 6.7	132.5–136.3	134.3 ± 6.6	132.4–136.6	− 0.1	< 0.001
Tr–Pn´	144.9 ± 7.0	142.9–146.9	144.9 ± 7.0	142.9–146.9	0.0	> 0.999	144.9 ± 7.0	142.9–146.9	145.3 ± 7.0	143.3–147.2	− 0.4	< 0.001	144.9 ± 7.0	142.9–146.9	145.8 ± 7.0	143.8–147.8	− 0.9	< 0.001
Tr–Sn	132.2 ± 6.4	130.4–134.0	132.2 ± 6.5	130.4–134.1	0.0	0.120	132.2 ± 6.4	130.4–134.0	132.3 ± 6.4	130.4–134.1	− 0.1	0.667	132.2 ± 6.4	130.4–134.0	132.3 ± 6.4	130.5–134.1	− 0.1	0.003
Scal'r–Scal'l	27.5 ± 3.6	26.5–28.5	27.5 ± 3.6	26.5–28.5	0.0	> 0.999	27.5 ± 3.6	26.5–28.5	27.5 ± 3.6	26.5–28.5	− 0.3	0.175	27.5 ± 3.6	26.5–28.5	27.5 ± 3.7	26.5–28.6	0.0	0.012
**Face lower third**																		
Pog´–B´–Li	133.2 ± 11.4	130.0–136.5	133.2 ± 11.5	130.0–136.5	0.0	> 0.999	133.2 ± 11.4	130.0–136.4	133.0 ± 11.5	129.7–136.2	− 0.2	0.005	133.2 ± 11.5	129.9–136.4	132.6 ± 11.8	129.3–136.0	0.6	< 0.001
E-Line–Li	3.5 ± 2.6	2.8–4.2	3.5 ± 2.6	2.8–4.3	0.0	> 0.999	3.6 ± 2.6	2.9–4.4	3.7 ± 2.6	2.9–4.4	− 0.1	> 0.999	3.9 ± 2.7	3.1–4.6	3.9 ± 2.7	3.1–4.6	0.0	> 0.999
Tr–Li	137.0 ± 6.8	135.1–138.9	137.0 ± 6.8	135.1–138.9	0.0	> 0.999	136.5 ± 6.8	134.6–138.4	136.5 ± 6.7	134.6–138.4	0.0	> 0.999	135.7 ± 6.7	133.8–137.6	135.8 ± 6.7	133.9–137.7	− 0.1	0.001
Tr–Pog'	142.6 ± 7.6	140.4–144.7	142.7 ± 7.6	140.4–144.7	0.1	> 0.999	141.8 ± 7.6	139.6–143.9	141.9 ± 7.7	139.6–143.9	− 0.1	> 0.999	140.6 ± 7.5	138.5–142.7	140.7 ± 7.5	138.6–142.8	− 0.1	0.067

*SD* standard deviation, *CI* confidence interval, *MD* mean difference.

**Table 6:** Mean values, SDs and 95% confidence intervals of the angular and linear changes in the middle and lower third of the face after mandibular surgery simulation with the corresponding p values of the comparison between the outcome of the surgical planning program Dolphin and ProPlan depending on the different displacement distances for mandibular advancement.

	Displacement distance																							
	0 mm						+ 4 mm						+ 7 mm						+ 10 mm					
	Dolphin imaging		ProPlan CMF		MD	p-value	Dolphin imaging		ProPlan CMF		MD	p-value	Dolphin imaging		ProPlan CMF		MD	p-value	Dolphin imaging		ProPlan CMF		MD	p-value
	Mean (SD)	95% CI	Mean (SD)	95% CI			Mean (SD)	95% CI	Mean (SD)	95% CI			Mean (SD)	95% CI	Mean (SD)	95% CI			Mean (SD)	95% CI	Mean (SD)	95% CI		
**Face middle third**																								
Ns–Sn–Ls	127.5 ± 8.2	125.2–129.8	127.7 ± 8.2	125.4–130.0	− 0.2	> 0.999	127.1 ± 8.1	124.8–129.4	126.6 ± 8.3	124.2–128.9	0.5	0.003	126.6 ± 8.1	124.3–128.9	125.7 ± 8.4	123.3–128.1	0.9	< 0.001	126 ± 8.0	123.3–128.1	124.9 ± 8.4	122.5–127.3	1.1	< 0.001
E-Line–Ls	5.8 ± 2.9	5.0–6.6	5.8 ± 2.9	5.0–6.6	0.0	> 0.999	7.4 ± 2.9	6.6–8.2	7.0 ± 2.9	6.2–7.8	0.4	< 0.001	8.6 ± 2.9	7.7–9.4	7.9 ± 2.9	7.1–8.7	0.7	< 0.001	9.7 ± 2.9	8.9–11.0	8.8 ± 2.9	8.0–9.6	0.9	< 0.001
Tr–Ls	134.7 ± 6.7	132.8–136.6	134.6 ± 6.7	132.8–136.5	0.1	> 0.999	134.8 ± 6.7	132.9–136.7	135 ± 6.7	133.1–136.9	− 0.2	< 0.001	134.9 ± 6.7	133.0–136.8	135.3 ± 6.7	133.4–137.2	− 0.4	< 0.001	135.1 ± 6.7	133.2–137.0	135.5 ± 6.8	133.6–137.4	− 0.4	< 0.001
Tr–Pn´	144.9 ± 7.0	142.9–146.9	144.9 ± 7.0	142.9–146.9	0.0	> 0.999	144.9 ± 7.0	142.9–146.9	144.9 ± 7.0	142.9–146.9	0.0	> 0.999	144.9 ± 7.0	142.9–146.9	144.9 ± 7.0	142.9–146.9	0.0	> 0.999	144.9 ± 7.0	142.9–146.9	144.9 ± 7.1	142.9–146.9	0.0	< 0.001
Tr–Sn	132.2 ± 6.4	130.4–134.0	132.2 ± 6.4	130.4–134.1	0.0	0.120	132.2 ± 6.4	130.4–134.0	132.3 ± 6.5	130.4–134.1	− 0.1	0.047	132.2 ± 6.4	130.4–134.1	132.3 ± 6.4	130.4–134.1	− 0.1	< 0.001	132.2 ± 6.4	130.4–134.1	132.3 ± 6.5	130.4–134.2	− 0.1	< 0.001
Scal'r–Scal'l	27.5 ± 3.6	26.5–28.5	27.5 ± 3.6	26.5–28.5	0.0	> 0.999	27.5 ± 3.6	26.5–28.5	27.5 ± 3.5	26.5–28.5	0.0	> 0.999	27.5 ± 3.6	26.5–28.5	27.5 ± 3.5	26.4–28.5	0.0	0.469	27.5 ± 3.6	26.5–28.5	27.4 ± 3.5	26.4–28.4	0.0	0.041
**Face lower third**																								
Pog´–B´–Li	133.2 ± 11.4	130.0–136.5	133.2 ± 11.5	130.0–136.5	0.0	> 0.999	133.2 ± 11.3	130.0–136.4	138.5 ± 11	135.4–141.6	− 5.3	< 0.001	133.3 ± 11.5	130.0–136.6	142.5 ± 11.0	139.4–145.6	− 9.2	< 0.001	133.3 ± 11.5	130.1–136.6	146.3 ± 11.1	143.1–149.4	− 13	< 0.001
E-Line–Li	3.5 ± 2.6	2.8–4.2	3.5 ± 2.6	2.8–4.3	0.0	> 0.999	2.9 ± 2.1	2.3–3.5	3.7 ± 2.6	2.9–4.4	− 0.8	< 0.001	2.6 ± 1.8	2.0–3.1	3.8 ± 2.7	3.0–4.6	− 1.2	< 0.001	2.5 ± 1.6	2.0–2.9	3.9 ± 2.8	3.1–4.6	− 1.4	< 0.001
Tr–Li	137.0 ± 6.8	135.1–138.9	137.0 ± 6.8	135.1–138.9	0.0	> 0.999	140.2 ± 6.8	138.3− 142.1	138.8 ± 6.8	136.8–140.7	1,4	< 0.001	142.6 ± 6.8	140.6–144.5	140.2 ± 6.9	138.1–142.1	2.4	< 0.001	145.0 ± 6.8	143.1–146.9	141.6 ± 6.9	139.6–143.5	3.4	< 0.001
Tr–Pog'	142.7 ± 7.6	140.4–144.7	142.6 ± 7.6	140.4–144.7	0.1	> 0.999	145.6 ± 7.6	143.4–147.7	145.4 ± 7.6	143.2–147.6	0.2	0.007	147.8 ± 7.6	145.7–150.0	147.5 ± 7.6	145.4–149.7	0.3	< 0.001	150.1 ± 7.5	148.0–152.3	149.7 ± 7.6	147.5–151.8	0.4	< 0.001

*SD* standard deviation, *CI* confidence interval, *MD* mean difference.

**Table 7:** Mean values, SDs and 95% confidence intervals of the angular and linear changes in the middle and lower third of the face after mandibular surgery simulation with the corresponding p values of the comparison between the outcome of the surgical planning program Dolphin and ProPlan depending on the different displacement distances for mandibular setback.

	Displacement distance																	
	0 mm						− 4 mm						− 7 mm					
	Dolphin imaging		ProPlan CMF		MD	p-value	Dolphin imaging		ProPlan CMF		MD	p-value	Dolphin imaging		ProPlan CMF		MD	p-value
	Mean (SD)	95% CI	Mean (SD)	95% CI			Mean (SD)	95% CI	Mean (SD)	95% CI			Mean (SD)	95% CI	Mean (SD)	95% CI		
**Face middle third**																		
Ns–Sn–Ls	127.5 ± 8.2	125.2–129.8	127.7 ± 8.2	125.4–130.0	− 0.2	> 0.999	128.2 ± 8.3	125.8–130.5	128.7 ± 8.1	126.4–131.1	− 0.5	0.002	128.5 ± 8.2	126.2–130.9	129.6 ± 8.1	127.3–131.9	− 1.1	< 0.001
E-Line–Ls	5.8 ± 2.9	5.0–6.6	5.8 ± 2.9	5.0–6.6	0.0	> 0.999	4.4 ± 2.7	3.6–5.2	4.7 ± 2.8	3.9–5.5	− 0.3	0.03	3.5 ± 2.4	2.8–4.2	4.0 ± 2.6	3.3–4.7	− 0.5	< 0.001
Tr–Ls	134.7 ± 6.7	132.8–136.6	134.6 ± 6.7	132.8–136.5	0.0	> 0.999	134.5 ± 6.7	132.6–136.4	134.3 ± 6.7	132.4–136.2	0.2	< 0.001	134.4 ± 6.7	132.5–136.3	134.1 ± 6.6	132.2–136.0	0.3	< 0.001
Tr–Pn´	144.9 ± 7.0	142.9–146.9	144.9 ± 7.0	142.9–146.9	0.0	> 0.999	144.9 ± 7.0	142.9–146.9	144.9 ± 7.0	142.9–146.9	0.0	.027	144.9 ± 7.0	142.9–146.9	144.9 ± 7.0	142.9–146.9	0.0	0.002
Tr–Sn	132.2 ± 6.4	130.4–134.0	132.2 ± 6.4	130.4–134.1	0.0	0.120	132.2 ± 6.4	130.4–134.0	132.2 ± 6.5	130.4–134.0	0.0	> 0.999	132.2 ± 6.4	130.4–134.1	132.2 ± 6.4	130.3–134.0	0.0	< 0.001
Scal'r–Scal'l	27.5 ± 3.6	26.5–28.5	27.5 ± 3.6	26.5–28.5	0.0	> 0.999	27.5 ± 3.5	26.5–28.5	27.5 ± 3.5	26.5–28.5	0.0	0.253	27.5 ± 3.6	26.5–28.5	27.5 ± 3.6	26.5–28.5	0.0	0.011
**Face lower third**																		
Pog´–B´–Li	133.2 ± 11.4	130.0–136.5	133.2 ± 11.5	130.0–136.5	0.0	> 0.999	133.1 ± 11.3	129.9–136.3	128.0 ± 11.8	124.7–131.4	5.1	< 0.001	133.1 ± 11.3	129.8–136.3	124.6 ± 11.9	121.2–128.0	8.5	< 0.001
E-Line–Li	3.5 ± 2.6	2.8–4.2	3.5 ± 2.6	2.8–4.2	0.0	> 0.999	4.4 ± 2.9	3.6–5.2	3.4 ± 2.4	2.7–4.1	1.0	< 0.001	5.1 ± 3.0	4.3–6.0	3.3 ± 2.3	2.6–3.9	1,8	< 0.001
Tr–Li	137.0 ± 6.8	135.1–138.9	137.0 ± 6.8	135.1–138.9	0.0	> 0.999	133.9 ± 6.8	131.9–135.8	135.3 ± 6.8	133.4–137.2	− 1.4	< 0.001	131.5 ± 6.8	129.6–133.5	134.1 ± 6.8	132.1–136.0	− 2,6	< 0.001
Tr–Pog'	142.6 ± 7.6	140.4–144.7	142.6 ± 7.6	140.4–144.7	0.1	> 0.999	139.6 ± 7.7	137.4–141.8	139.7 ± 7.7	137.6–141.9	− 0.1	0.145	137.4 ± 7.7	135.2–139.6	137.7 ± 7.7	135.5–139.9	− 0.3	< 0.001

*SD* standard deviation, *CI* confidence interval, *MD* mean difference.

**Table 8:** Mean values, SDs and 95% confidence intervals of the angular and linear changes in the middle and lower third of the face after bi-maxillary surgery simulation with the corresponding p-values of the comparison between the outcome of the surgical planning program Dolphin and ProPlan depending on the different displacement distances for maxillary advancement ( +) and mandibular setback (−).

	Displacement distance (maxillary advancement/ mandibular setback)																					
	0 mm									+ 3/−4 mm						+ 5/−4 mm						
	Dolphin imaging		ProPlan CMF				MD		p-value	Dolphin imaging		ProPlan CMF		MD	p-value	Dolphin imaging		ProPlan CMF			MD	p-value
	Mean (SD)	95% CI	Mean (SD)		95% CI					Mean (SD)	95% CI	Mean (SD)	95% CI			Mean (SD)	95% CI	Mean (SD)	95% CI			
**Face middle third**																						
Ns–Sn–Ls	127.5 ± 8.2	125.2–129.8	127.7 ± 8.2		125.4–130.0		− 0.2		> 0.999	123.4 ± 8.5	120.9–125.8	129.9 ± 8.3	127.5–132.2	− 6.5	< 0.001	120.2 ± 8.7	117.7–122.6	129.5 ± 8.3	127.1–131.8		− 9.7	< 0.001
E-Line–Ls	5.8 ± 2.9	5.0–6.7	5.8 ± 2.9		5.0–6.7		0.0		> 0.999	3.1 ± 2.2	2.4–3.7	3.5 ± 2.5	2.8–4.1	− 0.4	0.263	2.3 ± 2.0	1.7–2.8	2.9 ± 2.0	2.3–3.4		− 0.6	0.046
Tr–Ls	134.7 ± 6.7	132.8–136.6	134.6 ± 6.7		132.8–136.5		0.0		> 0.999	136.3 ± 6.7	134.4–138.3	136.2 ± 6.6	143.3–138.0	0.1	< 0.001	137.6 ± 6.7	135.7–139.5	137.3 ± 6.6	135.4–139.1		0.3	< 0.001
Tr–Pn´	144.9 ± 7.0	142.9–146.9	144.9 ± 7.0		142.9–146.9		0.0		> 0.999	145.4 ± 7.0	143.4–147.4	145.9 ± 7.0	143.8–147.9	− 0.5	< 0.001	145.8 ± 7.0	143.8–147.8	146.8 ± 7.0	144.8–148.8		− 1.0	< 0.001
Tr–Sn	132.2 ± 6.4	130.4–134.0	132.2 ± 6.5		130.4–134.1		0.0		0.120	132.9 ± 6.4	131.1–134.7	134.0 ± 6.4	132.2–135.8	− 1.1	< 0.001	133.4 ± 6.4	131.6–135.3	135.3 ± 6.4	133.4–137.1		− 1.9	< 0.001
Scal'r–Scal'l	27.5 ± 3.6	26.5–28.5	27.5 ± 3.6		26.5–28.5		0.0		> 0.999	27.4 ± 3.5	26.4–28.4	27.8 ± 3.6	26.8–28.8	− 0.4	< 0.001	27.5 ± 3.6	26.4–28.5	28.1 ± 3.6	27.1–29.2		− 0.6	< 0.001
**Face lower third**																						
Pog´–B´–Li	133.2 ± 11.4	130.0–136.5	133.2 ± 11.5		130.0–136.5		0.0		> 0.999	133.1 ± 11.3	129.9–136.3	124.8 ± 11.9	121.4–128.1	8.3	< 0.001	133.1 ± 11.4	129.8–136.3	122.7 ± 11.9	118.9–125.6	10.4		< 0.001
E-Line–Li	3.5 ± 2.6	2.8–4.2	3.5 ± 2.6		2.8–4.3		0.0		> 0.999	4.5 ± 2.9	3.7–5.3	3.0 ± 2.1	2.4–3.6	1.5	< 0.001	4.8 ± 3.0	3.9–5.6	2.9 ± 2.0	2.3–3.4	1.9		< 0.001
Tr–Li	137.0 ± 6.8	135.1–138.9	137.0 ± 6.8		135.1–138.9		0.0		> 0.999	133.9 ± 6.8	132.0–135.8	136.2 ± 6.7	134.3–138.1	− 2.3	< 0.001	133.4 ± 6.8	131.4–135.3	136.4 ± 6.7	134.4–138.1	− 3.0		< 0.001
Tr–Pog'	142.6 ± 7.6	140.4–144.7	142.7 ± 7.6		140.4–144.7		0.1		> 0.999	139.6 ± 7.7	137.5–141.8	139.7 ± 7.6	137.6–141.9	− 0.1	> 0.999	138.9 ± 7.6	136.7–141.0	139.1 ± 7.5	136.9–141.0	− 0.2		> 0.999

*SD* standard deviation, *n.s.* not significant, *CI* confidence interval, *MD* mean difference.

### Maxillary advancement

In the middle face, except for the distance Tr-Ls, all the measurements significantly differed between the two programs, even for the 3-mm maxillary advancement. However, the behaviour of the soft tissue was similar except for the nasolabial sulcus (Ns-Sn-Ls) that showed a diverging behaviour by increasing in ProPlan (0 mm: 127.7° ± 8.2°, 3 mm: 128.8° ± 8.2° and 5 mm: 129.5° ± 8.4°) and decreasing in Dolphin (0 mm: 127.5° ± 8.2°, 3 mm: 123.1° ± 8.4° and 5 mm: 119.9° ± 8.6°), and the nose width (Scal′l–Scal′r), which was almost unchanged in Dolphin (0 mm: 27.5 ± 3.6 mm, 3 mm: 27.5 ± 3.5 mm and 5 mm: 27.5 ± 3.6 mm) but increased in ProPlan (0 mm: 27.5 ± 3.6 mm, 3 mm: 27.8 ± 3.6 mm and 5 mm: 28 ± 3.6 mm).

In the lower third of the face, fewer changes were noted for the Dolphin software. Except for Tr–Pog′, the changes in the ProPlan software were significant compared with those in Dolphin for both advancement distances, especially for the mentolabial angle (Dolphin, 0 mm: 133.2° ± 11.4°, 3 mm: 133.2° ± 11.4° and 5 mm: 133.2° ± 11.4° vs. ProPlan, 0 mm: 133.2° ± 11.5°, 3 mm: 129.1° ± 11.4° and 5 mm: 126.8° ± 11.6°).

### Maxillary impaction

The measured changes were less compared with the maxillary advancement. Nevertheless, significant differences between the two programs were found, especially after the 5-mm impaction. In addition, a similar divergence was observed with regard to the nasolabial angle (Dolphin, 0 mm: 127.5° ± 8.2°, 2 mm: 123.1° ± 8.4° and 5 mm: 119.9° ± 8.6° vs. ProPlan, 0 mm: 127.7° ± 8.2°, 2 mm: 128.8° ± 8.2° and 5 mm: 129.5° ± 8.4°). Overall, the lower third of the face appeared to be only slightly altered by impaction in both programs.

### Mandibular advancement and setback

The alterations in the middle third of the face were comparable between the two programs, in spite of the resulting significant differences, especially after mandibular advancement. Tr–Pn, Tr–Sn and Scal′l–Scal′r changed only slightly compared with their baseline values, while Ns–Sn–Ls decreased and E-Line–LS and Tr–LS increased with increasing mandibular advancement and vice versa, with the corresponding setback.

On the other hand, significant differences were found between the two programs for the lower third of the face. For example, the mentolabial sulcus (Pog′–B′–Li) remained almost constant in the Dolphin software (range, − 7 to 10 mm: 133.1° ± 11.3° to 133.3° ± 11.5°), whereas this angle in the ProPlan software became smaller after the setback and larger after mandibular advancement (range, − 7 to 10 mm: 124.6° ± 11.9° to 146.3° ± 11.1°). Furthermore, the soft tissue behaviour was also divergent for E-Line-Li. While this distance in ProPlan became slightly smaller during mandibular setback and slightly larger during mandibular advancement (range, − 7 to 10 mm: 5.1 ± 3.0 mm to 2.5 ± 1.6 mm), it increased significantly during setback and decreased according to the advancement (range, − 7 to 10 mm: 3.3 ± 2.3 mm to 3.9 ± 2.8 mm). All differences were statistically significant. Tr-Li and Tr-Pog' both decreased during mandibular setback and increased during mandibular advancement. However, in the Dolphin software, this behaviour was more pronounced for Tr-Li (range, − 7 to 10 mm; Dolphin: 131.5 ± 6.8 mm to 145 ± 6.8 mm vs. ProPlan: 134.1 ± 6.8 mm to 141.6 ± 6.9 mm).

### Bi-maxillary procedure (maxillary advancement and setback)

The soft tissue behaved similarly to the simulation for maxillary advancement, but in some cases, the effects were more pronounced. Thus, the soft tissue prognosis after bi-maxillary surgery also diverged between the two programs for Ns-Sn-Ls (Dolphin, 0 mm: 127.5° ± 8.2°, + 3/ − 4 mm: 123.4° ± 8.5° and + 5/ − 4 mm: 120.2° ± 8.7° vs. ProPlan: 0 mm 127.7° ± 8.2°, + 3/ − 4 mm: 129.9° ± 8.3° and + 5/ − 4 mm: 129.9° ± 8.3°) and the nose width, which remained constant in Dolphin but increased in ProPlan (Dolphin, 0 mm: 27.5 ± 3.6 mm, + 3/ − 4 mm: 27.4 ± 3.5 mm and + 5/ − 4 mm: 27.5 ± 3.6 mm vs. ProPlan: 0 mm 27.5 ± 3.6 mm, + 3/ − 4 mm: 27.8 ± 3.6 mm and + 5/ − 4 mm: 28.1 ± 3.6 mm)*.* Otherwise, the simulation results of the two programs for the middle third of the face were similar, but Tr-Pn and Tr-Sn increased more in ProPlan.

The soft tissue prognosis in the lower third of the face was also similar. The mentolabial angle (Dolphin, 0 mm: 133.2° ± 11.4°, + 3/ − 4 mm: 133.1° ± 11.3° ± 8.5° and + 5/ − 4 mm: 133.1° ± 11.4° ± 8.7° vs. ProPlan: 0 mm: 133.2° ± 11.5°, + 3/ − 4 mm: 124.8° ± 11.9° and + 5/ − 4 mm: 122.7° ± 11.9°) and E-Line-Li (Dolphin, 0 mm: 3.5 ± 2.6 mm, + 3/ − 4 mm: 4.5 ± 2.9 mm and + 5/ − 4 mm: 4.8 ± 3.0 mm vs. ProPlan, 0 mm: 3.5 ± 2.6 mm, + 3/ − 4 mm: 3.0 ± 2.1 mm and + 5/ − 4 mm: 2.9 ± 2.0 mm) showed an even more pronounced divergent soft tissue behaviour. By contrast, Tr-Li and Tr-Pog′ decreased in both groups. However, the difference between the two programs was statistically significant for Tr-Li.

## Discussion

The aim of this study was to investigate the difference in three-dimensional soft tissue prediction between the two established software programs, Dolphin Imaging and ProPlan CMF, after different orthognathic surgical plans were made, varying in surgical technique and displacement distance. Possible differences should be determined by established linear and angular measurements, as these may be more relevant or more comprehensible from a clinical point of view than, for example, the use of conformed meshes. Only a computer-based study design as in the present study allows multiple operations in the same patient^19^.

In the present investigation, equal initial conditions were determined, as no significant differences in the initial situation before segment displacement were found between the two programs for all the measured parameters. To avoid deviations in the displacement distance, the models were aligned identically to the occlusal plane. The individual segments were then displaced congruently in both programs with adjustable controllers. Nevertheless, some possible sources of error still exist. These concern possible differences between the two programs with regard to the surgical planning tools as well as the manual landmark placement. In order to keep these as low as possible, the entire planning and simulation was carried out by only one investigator.

The present study was based only on a virtual study design; thus, comparison with real post-operative results is not possible or only possible to a limited extent. In this context, the tragus was selected as the fixed point for standardised measurement because it is a stable landmark^16–18^. The main focus of the study was the possibility of a difference in soft tissue simulation between two established programs.

Currently, only a few studies have addressed the accuracy of virtual soft tissue prognosis in the context of orthognathic surgery. Generally, the Dolphin and ProPlan programs are used for this purpose, but the use of Dolphin Imaging seems to be more widespread^9,15,20,21^. The numbers of cases are comparatively small, and the results are sometimes controversial. In this context, Petermann et al. found that Dolphin promises a good prediction of soft tissue behaviour in the sagittal plane, whereas Ahmad Akhoundi et al. described the opposite^20,21^.

Ahmad Akhoundi et al. examined and compared the ability and reliability of digitisation using Dolphin with conventional manual techniques and compared orthognathic prediction with actual outcomes^21^. They reported that the nasal tip presented the least predicted error and highest reliability. The least accurate regions were the subnasale and upper lip, and subnasale and pogonion. The authors concluded that this method of image prediction was suitable for patient education and communication. Peterman et al. also addresses the accuracy of soft tissue simulation prediction for bi-maxillary surgery in class III patients using the Dolphin software on cephalometric radiographs^20^. They reported that the pronasal point with deviations of 0.5 mm was the most accurate, the lip predictions were the most inaccurate. Peterman et al. explained the inaccuracy of lower lip prediction due to the effect of the anterior tooth position on the lower lip and muscle tone of the perioral muscles^20^.

Nadjmi et al. investigated the soft tissue prediction by the programs, Dolphin and Maxilim, on lateral cephalograms from pre- and post-operative cone-beam CT scans of 13 patients^9^. Afterwards, soft tissue landmarks set in post-operative photographs and pre-operative predictions were compared. For the vertical and horizontal positions of the upper lip, lower lip and nose, they found an accuracy of 70% for both programs and 50% in the chin area. They assumed that the high error rate in the chin was due to the mandibular autorotation.

The study of Knoops et al. was based on the data of 7 patients who achieved a maxillary advancement of 5.8 ± 1.2 mm. They compared the simulated outcomes of the ProPlan, Dolphin and another in-house software programs (PFEM) with the real post-operative outcome^15^. The authors found that Dolphin, whose simulations are based on a landmark-based algorithm, provides good results for cephalometric radiographs, but was limited with regard to three-dimensional accuracy. The simulation of the paranasal region using Dolphin was deficient compared with that using ProPlan. In general, ProPlan and PFEM provide better three-dimensional predictions with increasing bone segment shifts. Overall, a post-operative inaccuracy of approximately < 2 mm was found for the root mean square distance in all the programs^15^.

The present study showed a partially opposing soft tissue prediction between the two programs. Consequently, differences in comparison with clinical studies are also to be expected.

In the context of maxillary advancement, the change of the nasolabial sulcus diverged. In Dolphin, it decreased by 7.6° to 119.9°, and in ProPlan, it increased by 1.8° to 129.5°. This is probably attributed to the subnasal point, which changed mostly compared with the labium superius. In this context, Hellak et al. reported a reduction in nasolabial angle by − 6.65° ± 7.71° after a maxillary advancement of 5.5 mm^22^, and DeSesa et al. reported a change in subnasal point of 1.3 ± 1.8 mm after a 5.8 mm displacement^23^. Both studies demonstrated soft tissue simulations comparable with those by Dolphin. However, ProPlan simulated the opposite soft tissue behaviour. Significant differences were also found with regard to the submental fold. The soft tissue movements during maxillary advancement in Dolphin are almost limited to the directly associated soft tissues, whereas the lower part of the face remains unaffected. However, in ProPlan, the soft tissues of the lower part of the face also changed, but with decreasing intensity. In this context, Wermker et al. reported a further posterior position of the labium inferius of approximately 1.12 mm and the pogonion of approximately 0.89 mm for a maxillary advancement of 3.62 mm^24^. This contradicts the simulations of both programs. Regarding changes in nose width, Hellak et al. reported a significant widening of the nasal base of + 2.6 ± 1.3 mm after a maxillary advancement of 5.5 mm^22^. In general, they described a widening of the nose by half of the maxillary advancement. Results congruent to this were reported by DeSesa^23^. By contrast, in the present study, a slight tendency of widening of the nasal base was observed only in the prediction of ProPlan.

With regard to the simulated 2- and 5-mm maxillary impactions, comparison with clinical studies is difficult because generally, the impaction will be performed in combination with mandibular displacement^25–27^. Furthermore, the following autorotation is not taken into account in the software algorithms^21^. In this context, Steinhäuser et al. reported that depending on the type and extent of maxillary impaction, a significant advancement of the mandible in the pogonion point must be achieved^27^. Nevertheless, in the present study, impaction alone was simulated to exclude the potential influence of mandibular displacement. Only isolated differences were found between the two programs; moreover, the measurements were largely constant and mainly concerned the nasolabial angle and the distance of the labium superius to the E-line.

Regarding mandibular displacement, the effect on adjacent soft tissues to the bony displaced segment was very low in both programs, although ProPlan appeared to consider adjacent soft tissue changes more than does Dolphin. Consequently, the pronasale and subnasale remained unchanged in Dolphin, whereas the labium superius, which is closest to the bony displaced segment, changed mostly. With a mandibular setback of 7 mm, the nasolabial angle changed more in ProPlan than in Dolphin. For example, it increases by 1.9° with a 7-mm setback (cf. Dolphin: 1°) and decreases by 2.8° with advancement (cf. Dolphin; 1.5°). The situation is different for the submental fold. In Dolphin, the labium inferius, B' point and pogonion were similar assign. Therefore, the submental angle also remained constant at 133.2° ± 0.1°. By contrast, in ProPlan, the greatest changes were found for the B' point and pogonion, and the least change was found in the lower lip area. The angle became 8.6° smaller with the 7-mm setback and 13.1° larger with the 10-mm advancement. Stern et al. investigated the soft tissue changes after mandibular advancement surgery in class II patients^28^. They found that the soft and hard tissues of the chin moved forward and downward and the position of the upper lip remained unchanged, whereas the lower lip moved forward and upward and decreased in thickness, which led to a decrease in thickness and a small decrease of the depth of the mentolabial fold. These findings were also reported in former investigations^29–31^. In this context, recently, Möhlhenrich et al. investigated the effects of different surgical techniques and displacement distances on the soft tissue profile retrospectively^32^. They found that for the mandibular setback in class III patients, only a slight increase in nasolabial angle (up to 2.13° ± 4.78°) and distance Ls-E-Line (up to 1.69 ± 2.12 mm) occurred. In the lower face, they described a maximum decrease of − 15.80° ± 17.50° in the mentolabial angle and a maximum increase of 1.18 ± 4.55 mm in the distance Li-E-Line. Furthermore, they reported a maximum increase of 2.48° ± 6.99° in the nasolabial angle and a maximum decrease in mentolabial angle of 21.60° ± 18.80° during mandibular advancement in class II patients. With regard to the lip distances to the E-line, a maximum decrease of − 3.46 ± 2.33 mm in the Ls-E-Line and − 1.59 ± 2.94 mm in the Li-E-Line were found. Therefore, the results of the ProPlan prediction in the present study conformed more with these findings than those of Dolphin prediction.

The simulation results of the bi-maxillary planning were similar to those of the maxillary advancement. The nasolabial angle decreases by 7.3° in Dolphin but increases by 2.2° in ProPlan. Both predictions for this angle were less than those in the study of Ghassemi et al., who reported a decrease in average of 9.5°^33^. Similar results were described by Park et al.^34^. However, Al-Gunaid et al. found that after a 2.3-mm maxillary advancement combined with a 5.5-mm mandibular setback, an increase of approximately 6.0° in the nasolabial angle was observed^35^. Depending on the study considered, soft tissue prediction of the nasolabial angle is better in Dolphin or ProPlan. The submental angle in the present study remained unchanged in Dolphin owing to the equal displacement of the three landmarks of approximately 3 mm posteriorly. However, in ProPlan, the angle was reduced by 8.4. In this context, Al-Gunaid et al. reported a reduction in submental angle of approximately 9°; and Marsan et al., approximately 4.9°. Therefore, both studies are consequently more in line with the prediction of ProPlan^35^. The nose width remained constant at nearly 27.5 mm in Dolphin but increased slightly in both maxillary simulations to approximately 0.3 and 0.6 mm in ProPlan. Thus, the results of ProPlan confirmed the results of the study of Hemmatpour et al.^36^. In the present study, after maxillary advancement of 4 mm and mandibular setback of 7 mm, the width of the nose widened by 0.4 mm.

## Conclusion

The present study shows that ideal soft tissue prediction in the context of orthognathic surgery is only possible to a limited extent. Differences exist between the simulated soft tissue behaviour and already postulated data in the current literature and between the programs, as demonstrated in this investigation. In this context, it must be critically noted that the presented significant differences in the range between 2 and 3 mm must not necessarily have to be clinically relevant.

Owing to design of the present study, no statement can be made regarding the preference of either program, as further clinical studies are necessary to develop a generally applicable algorithm. Therefore, informing patients about possible post-operative deviations from the pre-operative simulation it still a fundamental step.

## Data Availability

All data generated or analyzed during this study are included in this published article.
